# 

**Published:** 1857-05

**Authors:** 


### LIST OF COLLABORATORS.

Walter F. Atlee, M.D., Philadelphia.

John Bell, M.D., Philadelphia, formerly Professor of Medicine, Medical College of Ohio.

T. S. Bell, M.D., Louisville, Ky.

S. M. Bemiss, M.D., Louisville, Ky.

L. P. Blackburn, M.D., Natchez, Miss.

G. C. Blackman, M.D., Professor of Surgery, Medical College of Ohio.

W. S. Bowen, M.D., New York.

J. L. Bobbs, M.D., formerly Professor of Anatomy, Indianapolis.

W. M. Boling, M.D., Montgomery, Ala., formerly Professor of Midwifery, Transylvania University, Lexington, Ky.

N. Bozeman, M.D., Montgomery, Ala.

J. T. Bradford, M.D., Augusta, Ky.

John H. Brinton, M.D., Lecturer on Operative Surgery, Philadelphia.

W. S. Chipley, M.D., Professor of Medicine, Transylvania University.

A. Clapp, M.D., New Albany, Ia.

D. B. Cliffe, M.D., Franklin, Tenn.

J. Da Costa, M.D., Lecturer on the Practice of Medicine, at the Philad. Med. Association.

M. Dupierris, M.D., Havana, Cuba.

W. F. Edgar, M.D., United States Army.

S. C. Farrar, M.D., Jackson, Miss.

C. S. Fenner, M.D., Memphis, Tenn.

Austin Flint, M.D., Professor of Clinical Medicine and Pathology, University of Buffalo.

W. Y. Gadbury, M.D., Benton, Miss.

O. C. Gibbs, M.D., Chautauque County, New York.

Dr. J. P. Hall, Glasgow, Ky.

John Hardin, M.D., Professor of Obstetrics, Kentucky School of Medicine, Louisville.

Edward Hartshorne, M.D., One of the Surgeons to Wills Hospital for Diseases of the Eye, Philadelphia.

E. B. Haskins, M.D., Clarksville, Tenn., late President of the Tennessee State Medical Society.

C. A. HENTZ, M.D., Marianna, Florida.

ADDINELL HEWSON, M.D., One of the Surgeons to Wills Hospital for Diseases of the Eye, Philadelphia.

SAMUEL L. HOLLINGSWORTH, M.D., late Editor of the Medical Examiner, Philadelphia.

WILLIAM A. HAMMOND, M.D., United States Army.

WILSON JEWELL, M.D., Philadelphia.

G. A. KUNKLER, M.D., Madison, Ia.

WM. V. KEATING, M.D., Lecturer on Obstetrics and the Diseases of Women, in the Philadelphia Medical Association.

BEN. LOGAN, M.D., Shreveport, La.

A. LOPEZ, M.D., Mobile, Ala., formerly President of the Alabama State Medical Society.

B. I. RAPHAEL, M.D., New York.

J. C. REEVE, M.D., Dayton, Ohio.

L. C. RIVES, M.D., formerly Professor of Midwifery, Medical College of Ohio.

J. LAWRENCE SMITH, M.D., Professor of Chemistry, University of Louisville.

W. C. SNEED, M.D., Frankfort, Ky., President, Kentucky State Medical Society.

C. H. SPILMAN, M.D., Harrodsburg, Ky., late President of Kentucky State Medical Society.

GEO. SUTTON, M.D., Aurora, Ia.

W. L. SUTTON, M.D., Georgetown, Ky., formerly President Kentucky State Medical Society.

HORATIO R. STORER, M.D., One of the Physicians to the Boston Lying-in Hospital, Boston, Mass.

S. THOMPSON, M.D., Albion, Ill.

E. WILLIAMS, M.D., Cincinnati, Ohio.

### Bi-Monthly Bibliographical Record.

### AMERICAN.

Statistical Report on the Sickness and Mortality in the Army of the United States, embracing a Period of Sixteen Years, from Jan., 1839, to Jan., 1855. Prepared under the direction of Brevet Brigadier General Thomas Lawson, Surgeon-General U. S. Army. By Richard H. Coolidge, M. D., Assistant Surgeon U. S. Army. 4to., pp. 690. Washington, 1856. (Received from the Author.)

Hints on the Medical Examination of Recruits for the Army, and on the Discharge of Soldiers from the Service on Surgeon's Certificate. Adapted to the Service of the United States. By Thomas Henderson, M. D., Assistant Surgeon U. S. Army. A New edition. Revised by R. H. Coolidge, M.D., Assist.-Surgeon U. S. Army. Philadelphia, J. B. Lippincott & Co., 1856. (Received from the Publishers.)

The Physician's Pocket Dose and Symptom Book. By Joseph H. Wythes, A.M., M.D., second edition. Lindsay & Blakiston, Philadelphia, 1857. (Received from the Publishers.)

Transactions of the Fifth Annual Meeting of the Kentucky State Medical Society. Held in the City of Frankfort, on the 6th and 7th of February, 1856. Pamphlet.

Quarterly Summary of the Transactions of the College of Physicians of Philadelphia. January, 1857. Pamphlet.

Disease a Unit; or, Medicine a Science. By H. Backus, Selma, Alabama, 1857. Pamphlet, pp. 60. (Received from the Author.)

An Essay on the Relation of Bilious and Yellow Fever. Prepared at the request of and read before the Medical Society of the State of Georgia, at its session held at Macon, on the 9th of April, 1855. By Richard D. Arnold, M. D., Professor of the Theory and Practice of Medicine in the Savannah Medical College. Pamphlet, pp. 30. Illustrated with three colored Lithographs of the liver. (Received from the Author.)

Report of the Pennsylvania Hospital for the Insane, for the year 1856. By Thomas S. Kirkbride, M. D., Physician to the Institution. Philadelphia, 1857. Pamphlet, pp. 68. (Received from Dr. Kirkbride.)

Report of the Board of Managers of the Missouri State Lunatic Asylum, for the year 1856. Pamphlet, pp. 43.

Fourteenth Annual Report of the Managers of the New York State Lunatic Asylum, at Utica, 1857.

### FOREIGN.

Lettsomian Lectures on Insanity. By Forbes Winslow, M.D., D. C. L., late President of the Medical Society of London, etc. 8vo. pp. 160. John Churchill, London, 1857. (Received from the Author.)

Pathological Chemistry in its Applications to the Practice of Chemistry. Translated from the French of MM. Becquerel and Rodier. By Stanhope Templeman Speer, M.D., &c. &c. 8vo. pp. 560. London, 1857. (Received from the Translator.)

On the Constitutional Treatment of Female Diseases. By Edward Rigby, M. D., &c. &c. 12mo. pp. 250. London, 1856. Republished by Blanchard & Lea, Philadelphia, 1857. (Received from the Publishers.)

Clinical Lectures on Certain Diseases of the Urinary Organs and on Dropsies. By Robert Bentley Todd, M. D., F. R. S. Physician to King's College Hospital, London. Philadelphia, 8vo. pp. Republished by Blanchard & Lea, 1857. (Received from the Publishers.)

The Physiological Anatomy and Physiology of Man. By Robert Bentley Todd, M. D., &c. &c., and William Bowman, F.R.S., &c. &c. Part IV, Sect. II (with title, preface, &c., completing the work), London. Philadelphia, 8vo. pp. 725. Republished by Blanchard & Lea. (Received from the Publishers.)

An Exposition of the Signs and Symptoms of Pregnancy, with some other papers on subjects connected with Midwifery. By W. F. Montgomery, A.M., M.D., Professor of Midwifery in King's & Queen's College of Physicians in Ireland, &c. &c., 8vo. pp. 540. Republished from the second London edition by Blanchard & Lea, Philadelphia, 1857. (Received from the Publishers.)

Reflections on Petit's Operation, and on Purgatives after Herniotomy. By Joseph Sampson Gamgee, Assistant Surgeon to the Royal Free Hospital. Pamphlet, pp. 45. H. Balliere, London, 1855. (Received from the Author.)

Researches in Pathological Anatomy and Clinical Surgery. By Joseph Sampson Gamgee, Assistant Surgeon, &c. &c. 8vo. pp. 216. H. Balliere, London, 1856. (Received from the Author.)

